# Serum lipid profiles in patients with acute myocardial infarction in Hakka population in southern China

**DOI:** 10.1186/s12944-017-0636-x

**Published:** 2017-12-16

**Authors:** Zhixiong Zhong, Jing Liu, Bing Li, Cunren Li, Zhidong Liu, Min Yang, Qifeng Zhang, Wei Zhong, Pingsen Zhao

**Affiliations:** 1 Center for Cardiovascular Diseases, Meizhou People’s Hospital (Huangtang Hospital), Meizhou Hospital Affiliated to Sun Yat-sen University, Meizhou, 514031 People’s Republic of China; 2 Clinical Core Laboratory, Meizhou People’s Hospital (Huangtang Hospital), Meizhou Hospital Affiliated to Sun Yat-sen University, Meizhou, 514031 People’s Republic of China; 3 Center for Precision Medicine, Meizhou People’s Hospital (Huangtang Hospital), Meizhou Hospital Affiliated to Sun Yat-sen University, Meizhou, 514031 People’s Republic of China

**Keywords:** Dyslipidemia, Coronary heart disease, Myocardial infarction, Low-density lipoprotein cholesterol, High-density lipoprotein cholesterol, Total cholesterol, Cholesterol, Triglyceride

## Abstract

**Background:**

Little is known about serum lipid levels comparison of patients with acute myocardial infarction (AMI) in Hakka patients in southern China. To estimate the prevalence lipid profiles in Hakka patients with AMI in southern China.

**Method:**

We analyzed 1382 patients with a first AMI in Hakka patients in southern China between Jan. 2015 and Dec. 2015.

**Results:**

Our findings demonstrated that low-density lipoprotein cholesterol (LDL), total cholesterol (TC), and triglyceride (TG) were higher in nonelderly than in elderly for males. There were significant differences in TC, LDL, HDL, and TG among various age groups for both males and female patients (*P* < .05). TC, LDL, HDL, and TG were higher in females than males for the elderly, and the LDL levels of females were higher in 70–79,80–89 year age groups than males. The HDL level of female patients was higher than males in those 50–59, 60–69, and 70–79 year age groups. Compared with males, females had higher level of TG in the 60–69, 70–79, and 80–89 year age groups and had higher level of TC in the 50–59, 70–79, and 80–89 year age groups, respectively. Isolated high TG (normal LDL + normal HDL+ high TG) was most common type of combined dyslipidemia for female elderly (22.2%), female nonelderly (23.2%) and male elderly (24.1%) patients.

**Conclusion:**

Our results confirmed that serum lipid levels varied in age and gender in Hakka patients with acute myocardial infarction. Dyslipidemia is more prevalent in the non-elderly than in the elderly for males. Levels of TC, LDL, HDL, and TG were higher in females than males for the elderly Hakka population in southern China.

## Background

In recent years, cardiovascular disease (CVD) is the leading cause of mortality and morbidity worldwide in both male and female populations, that is widely accepted [1, 2]. Some clinical studies show that the incidence of coronary artery disease (CAD) continue to increase; about 1 in 5 Chinese is suffering from CAD [2].Cardiovascular disease is regarded as a multifactorial disease, which affected by the environment and genetic factors. Traditional cardiovascular risk factors, such as smoking, drinking, diabetes, dyslipidemia and advanced age, can increase the risk of cardiovascular disease. [3] Among the many cardiovascular disease risk factors, dyslipidemia is considered as the most important factor, which is a strong predictor for cardiovascular outcomes after AMI. Lipid profile evaluation including total cholesterol (TC), triglycerides (TG), low-density lipoprotein (LDL-C) and high-density lipoprotein (HDL-C) allows an assessment of CVD risk. A growing evidences indicates that elevated concentrations of TG, TC, LDL-C and decreased HDL-C accelerate the development of atherosclerotic plaques [4–7].

Dyslipidemia is an important risk factor for cardiovascular disease after acute myocardial infarction. Different age and gender have different lipid levels. Previous studies have shown age- and gender- differences in lipid profile in AMI patients in East China, and dyslipidemia is biased toward young people, at the same time, some researchers also reported the lipid metabolism in urban and rural areas of southern China [8].However, little is known about age and gender-related lipid levels comparison of patients with acute myocardial infarction (AMI) in the Hakka population of southern China. Understanding age-and gender-related differences in post-AMI lipid profiles and the characteristic of dyslipidemia in the Hakka population in southern China may provide important implications for treatment and guide strategy to reduce age- and gender-related differences in outcomes.

## Methods

### Subjects

This is a retrospective study. A total of 1382 patients with first myocardial infarction were collected through Jan. 2015 and Dec. 2015 in Meizhou People’s Hospital (Huangtang Hospital), Meizhou Hospital Affiliated to Sun Yat-sen University, China. The patients were excluded containing: (1) previous AMI; (2) Incomplete datas of lipid files; (3) Patients with chronic liver dysfunction, malignant tumors and other serious medical diseases; (4) Patients who were taking medications of lipid-lowering, such as statins and fibrates. The study approved by Human Ethics Committees of Meizhou People’s Hospital (Huangtang Hospital), Meizhou Hospital Affiliated to Sun Yat-sen University, Guangdong province, China. All the patients had signed the informed consent.

### Data collection and analysis

Data were prospectively collected, such as gender, age and diabetes, obesity, hypertension and family history. Peripheral venous blood samples were collected within 24 h after admission for measuring lipid profiles. Lipid profiles were tested by selective solubilization method (AU5400 analyzer, Beckman Coulter, CA, USA).The continuous variables and categorical variables were expressed as mean ± standard deviation, as %, or frequencies with percentage, respectively. IBM SPSS Statistics 21.0 (IBM, Armonk, NY, USA) was used for all statistical analyses.

Differences lipid profiles between groups based on were analyzed using χ^2^ test, Kruskal-Wallis test or One-way ANOVA analysis of variance test. A probability value of *P* < 0.05 was considered significant for this study.

## Results

### Clinical characteristics of Hakka patients with AMI

**Fig. 1:**
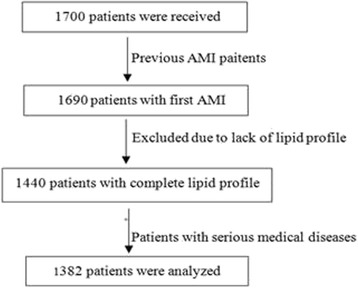
Flow chart of enrolled Patients

The flow chart of study are show in Fig. 1.Sample clinical baseline characteristics of the AMI patients were shown in Table 1. There were 1382 patients with AMI were enrolled in this study. The average age were similar between nonelderly and elderly. (55 years for men in nonelderly and 75 years for men in elderly, 57 years for women in nonelderly and 77 years for women in elderly). The rate of smoking and Hypertension were significantly difference between nonelderly and elderly for males.

**Table 1:** Clinical characteristics of Hakka patients with AMI

	Nonelderly		Elderly	
	Male	Female	Male	Female
Cases	485	86	586	225
Age (y)	55 ± 7	57 ± 6	75 ± 6	77 ± 7
BMI	22.4 ± 1.5	22.1 ± 2.1	22.4 ± 2.2	21.8 ± 1.7
Smoking, n (%)	251(51.8)	0(0)**	168(34.6)^##^	–
Alcoholdrinking, n (%)	13(2.7)	0(0)**	26(4.4)	–
Hypertension, n (%)	218(44.9)	52(60) **	319(54.4)^##^	132(22.5)**^##^
Diabetes, n (%)	137(28.2)	37(43)**	151(25.8)	71(31.5)^#^
CHD, n (%)	46(9.4)	8(9.3)	82(14)	28(12.4)
CVD, n (%)	27(5.5)	10(11.6) *	62(10.6)^##^	24(10.7)
TC (mmol/L)	4.88 ± 1.21	5.27 ± 1.72	4.58 + 1.15^##^	5.16 ± 1.67**
HDL (mmol/L)	1.25 ± 0.39	1.47 ± 0.399**	1.3 ± 0.4	1.43 ± 0.41**
LDL (mmol/L)	2.80 ± 0.83	2.83 ± 0.92	2.6 ± 0.84^##^	2.82 ± 0.97**
TG (mmol/L)	2.15 ± 1.6	2 ± 1.33	1.49 ± 1^##^	1.87 ± 1.44**
TC/HDL	4.06 ± 1.04	3.68 ± 1.06**	3.71 ± 1.01^##^	3.75 ± 1.11
LDL/HDL	2.38 ± 0.86	2.01 ± 1.72**	2.14 ± 0.83^##^	2.08 ± 0.84
TG/HDL	1.78 ± 1.18	1.48 ± 1.01*	1.27 ± 1.10^##^	1.38 ± 0.98

*AMI* acute myocardial infarction, *CHD* coronary heart disease, *CVD* cerebrovascular disease, *HDL* high-density lipoprotein cholesterol, *LDL* low-density lipoprotein cholesterol, *TC* total cholesterol, *TG* triglyceride

Compared with males, *P < .05 and **P < .01 for both the nonelderly and the elderly

Compared with the nonelderly, ^#^ *P* < .05 and ^##^ *P* < .01 for both males and females

Levels of TC, LDL, and TG were lower in elderly than in non-elderly for the males. Female of lipid profiles, which containing TC, LDL, HDL, and TG, were all significantly higher females than in males for the elderly. However, in the non-elderly group, there were no significant differences in TC, LDL, TG between male and female except HDL.

### Age and gender differences of LDL levels in Hakka patients with AMI

**Fig. 2:**
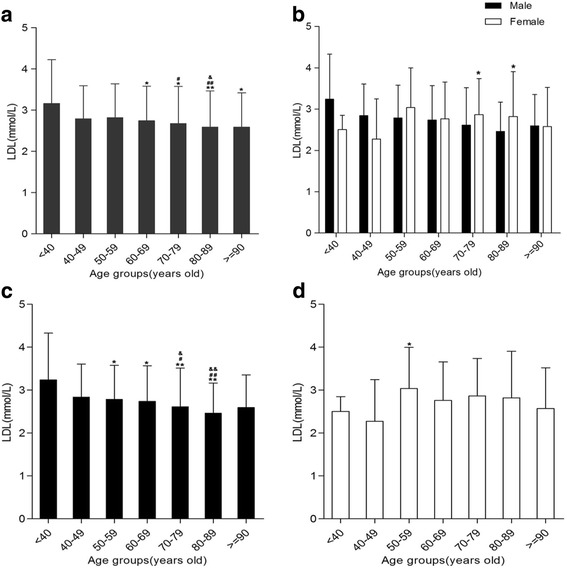
Low-density lipoprotein (LDL) levels in various age groups. **a** Comparison between <40 years of age group and more than 60 years of various age groups, respectively,*P < .05,**P < 0.01. Comparison between 50 and 59 year age groups and 70–79 and 80–89 year age groups, respectively, ^#^ *P* < .05,^##^ *P* < .01. Comparison between 60 and 69 year age groups and 80–89 year age groups were made, ^&^ *P* < .05. **b** Compared with males in the same age group, *P < .05. **c** For males, comparison between <40 years of age group and 50–89 years of various age groups, respectively, *P < .05, **P < .01. Comparison between 40 and 49 and 50–59 year age groups and 70–79 and 80–89 year age groups, respectively, ^#^ *P* < .05,^##^ *P* < .01.^&^ *P* < .05,^&&^ *P* < .01. **d** For females, Comparison between 40 and 49 and 50–59 year age group, *P < .05

The levels of LDL in different age groups were statistically significant (*P* < .05 or <.01, Fig. 2a) and females have higher LDL in both 70–79 (males vs. females, 2.61 ± 0.90 vs. 2.86 ± 0.87, *P* < .05) and 80–89(males vs females, 2.46 ± 0.70 vs. 2.82 ± 0.70, *P* < .05) year age groups compare to males (Fig. 2b). For male patients, LDL levels in <40 year age group was higher than those in 50–89 year age groups. At the same time, 40–49 and 50–59 year age groups were higher LDL levels compare to 70–79 and 80–89 year age groups (Fig. 2c). However, 50–59 year age group of LDL levels was higher than 40–49 year age group for female patients (Fig. 2d).

### Age and gender differences of HDL levels in Hakka patients with AMI

**Fig. 3:**
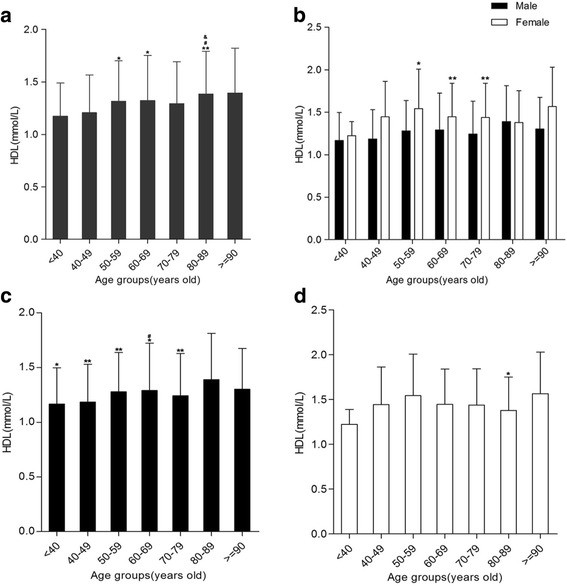
High-density lipoprotein (HDL) levels in various age groups. **a** Comparison between 40 and 49 year of age group and 50–59,60–69,80–89 years of various age groups, respectively, *P < .05,**P < .01. Comparison between <40 and 80–89 year age groups were made, ^#^ *P* < .05. Comparison between 70 and 79 year age groups and 80–89 year age groups were made, ^&^ *P* < .05. **b** The proportions of acute myocardial infarction patients who had high-density lipoprotein (HDL) levels. **c** For males, comparison between 80 and 89 year of age group and less than 79 years of various age groups, respectively, *P < .05,**P < .01. Comparison between 40 and 49 and 60–69 year age groups were made, ^#^ *P* < .05. **d** For females, comparison between 50 and 59 and 80–89 year age group. *P < .05

The levels of HDL in different age groups were statistically significant (*P <* .05 or *<*.01, Fig. 3a). The HDL levels of females in 50–59, 60–69, and 70–79 year age groups were higher than in males, respectively (Fig. 3b).Variance analysis showed that HDL levels in 80–89 year age group was higher than those ≤79 year-old year age groups for male patients (Fig. 3c). Similarly, The data implicated HDL level only in 80–89 year age group was lower compare to 50–59 year age group for females patients (Fig. 3d).

### Age and gender differences of TG levels in Hakka patients with AMI

**Fig. 4:**
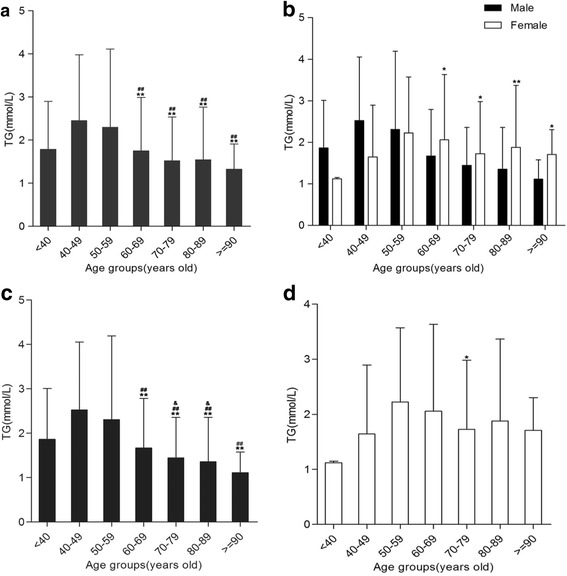
Triglyceride (TG) levels in various age groups. **a** Crossing comparisons of arbitrary two age groups between < 40, 40–49 year of age groups and more than 60 years of various age groups, respectively,*P < .05,**P < .01, ^#^ *P* < .05,^##^ *P* < .01. **b** Compared with males in the same age group, *P < .05, **P < .01. **c** For males, Crossing comparisons of arbitrary two age groups between 40 and 49,50–59 year of age groups and more than 60 years of various age groups, *P < .05,**P < .01, ^#^ *P* < .05,^##^ *P* < .01. Comparison between 60 and 69 and 70–79, 80–89 year age groups was made, ^&^ *P* < .05. **d** For females, Comparison between 50 and 59 and 70–79 year age groups was made,*P < .05

The levels of TG among various age groups were statistically significant for males and females AMI patients (*P* < .05 or <.01, Fig. 4a). Compared with males, females had higher level of TG in the 60–69, 70–79, and 80–89 year age groups respectively (Fig. 4b).Cross-comparison of arbitrary two age groups demonstrated 40–49 and50–59 year age groups were higher TG levels compare to in 60–69, 70–79, 80–89, and ≥90 year age groups, as well as 60–69 year age group was higher TG level compare with 70–79, 80–89 year age groups for males (Fig. 4c). Pair wise comparison implicated TG level only in 70–79 year age group was lower than that in 50–59 year age group for females AMI patients (Fig. 4d).

### Age and gender differences of TC levels in Hakka patients with AMI

**Fig. 5:**
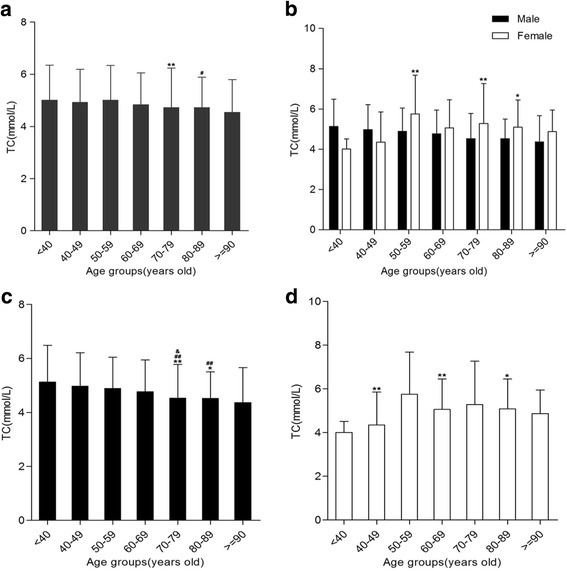
Total cholesterol (TC) levels in various age groups. **a** Comparison between 50 and 59 year of age group and 70–79,80–89 years of various age groups, respectively, *P < .05,**P < .01. **b** Compared with males in the same age group, *P < .05, **P < .01. **c** For males, Crossing comparisons of arbitrary 2 age groups between 40 and 49,50–59 year of age groups and 70–79, 80–89 years of age groups,*P < .05,**P < .01.^#^ *P <* .05,^##^ *P < .*01. Comparison between 50 and 59 and 70–79 year age groups, ^&^ *P < .*05. **d** For females, Comparison between 50 and 59 and 40–49,60–69,80–89 year age group, respectively,*P < .05,**P < .01

The levels of TC among various age groups were statistically significant for males and females AMI patients (*P* < .05 or <.01, Fig. 5a). The TC levels of females in the 50–59, 70–79, and 80–89 year age groups were higher compare to males, respectively (Fig. 5b). Cross-comparison of arbitrary two age groups demonstrated 40–49 and50–59 year age groups were higher TC levels compare to 70–79, 80–89 age groups, respectively, and 60–69 year age group was higher TC level compare with 70–79 year age groups for males (Fig. 5c). Pair wise comparison implicated TG level in 50–59 year age group was higher than those in 40–49, 60–69,80–89 year age groups, respectively, for females AMI patients (Fig. 5d).

### Comparison of lipid abnormalities in elderly and non-elderly Hakka patients with AMI

According to the Guideline for the Prevention and Treatment of Chinese Adult Dyslipidemia 2007, serum level of HDL <1.04 mmol/L (40 mg/dL) and ≥1.04 mmol/L (40 mg/dL) were regarded as low and normal, respectively. Serum level of LDL <3.37 mmol/L (130 mg/dL) and ≥3.37 mmol/L (130 mg/dL) were regarded as high and normal, respectively. Serum level of TC <5.18 mmol/L (200 mg/dL) and ≥5.18 mmol/L (200 mg/dL) were regarded as normal and high, respectively.; serum level of TG <1.70 mmol/L (150 mg/dL) and TG ≥1.70 mmol/L (150 mg/dL) were regarded as normal and high, respectively [9]. The distribution of dyslipidemia in AMI patients were shown in Table 2. The results indicated the most common type of combined dyslipidemia was isolated high TG (normal LDL+ normal HDL+ high TG) that reflect in female elderly (22.2%), female nonelderly (23.2%) and male elderly (24.1%). For males, the proportion of isolated high TG was higher in the non-elderly. In addition High LDL+ normal HDL + high TG, Categories of combined dyslipidemia were not statistically significant between the nonelderly and the elderly for females. Compare with the non-elderly females, males had lower the proportions of high LDL+ normal HDL + high TG is lower and higher the proportion of normal LDL+ low HDL+ high TG is higher for males. For the elderly, males had lower proportions of isolated high LDL, but proportion of isolated low HDL is higher than females. At the same time, the proportions of high TG were lower in the elderly than those in the nonelderly, while contrary to low HDL for males. Similarly, males had lower high LDL and high TG than females for elderly patients, while contrary to low HDL.

**Table 2:** Combined dyslipidemia in Hakka patients with AMI

	Male		Female	
	Nonelderly	Elderly	Nonelderly	Elderly
High LDL+ low HDL + high TG	10(2.3%)	7(1.2%)	1(1.2%)	2(0.88%)
High LDL+ low HDL+ normal TG	9(1.8%)	7(1.2%)	1(1.2%)	4(1.77%)
High LDL+ normal HDL + high TG	47(9.7%)	20(3.4%)*	15(17.4%)^##^	23(10.2%)**^##^
High LDL+ normal HDL+ normal TG	36(7.4%)	64(10.9%)**	6(7.0%)	29(12.9%)
Normal LDL+ low HDL+ high TG	58(12%)	48(8.2%)	1(1.2%)^##^	7(3.1%)^##^
Normal LDL+ low HDL+ normal TG	57(11.8%)	87(14.8%)**	7(8.1%)	13(5.7%)^##^
Normal LDL+ normal HDL+ normal TG	151(31.1%)	281(48.0%)**	35(40.6%)^##^	97(43.1%)^##^
Normal LDL+ normal HDL+ high TG	117(24.1%)	72(12.3%)**	20(23.2%)	50(22.2%) ^##^
Low HDL	89(18.6%)	149(25.4%)**	10(11.6%)	26(11.6%)^##^
High LDL	102(21.0%)	98(16.7%)	23(26.7%)	58(25.7%)^##^
High TG	208(42.9%)	147(25.1%)**	37(43.0%)	82(36.4%)^##^

*AMI* acute myocardial infarction, *HDL* high-density lipoprotein cholesterol, *LDL* low-density lipoprotein cholesterol, *TG* triglyceride

Compared with the nonelderly of the same sex, *P < .05 and **P < .01

Compared with females of the same age group, ^*##*^ *P <* .01

## Discussion

Lipid abnormality is one of important risk factor for cardiovascular disease in AMI patients. In particular, LDL-C and HDL-C are important factor for atherosclerosis and cardiovascular disease development [1]. More and more evidences that a decrease in LDL-C levels or a increase in HDL levels can prevent the occurrence of cardiovascular disease [4]. This study was aimed at investigating serum lipid levels in AMI patients in southern China. Our research show that, although all enrolled patients were Hakka population (Han Chinese immigrated from northern China to southern China hundreds or thousand years ago) and came from the same geographical region (Meizhou, a most populated city with Hakka peoples in China), different age and gender subgroups had different lipid abnormality patterns.

Compared with the elderly, the nonelderly have higher levels of LDL for males. In addition, data indicated that prevalence of combined dyslipidemia was higher in non-elderly (59.4–68.9%) than in elderly (52–56.9%). It indicated that dyslipidemia was more prevalent among the nonelderly than the elderly. Therefore, it was necessary for the nonelderly to accepted lipid-lowering therapy and choose more effective lipid-lowering drugs. TG levels in nonelderly were higher compared to elderly for both males and females in south China. Compare with males, females had higher levels of TC and TG in both 50–59, 60–69 year age groups, respectively, which were considered as the perimenopausal window for women. This results was inconsistent with the conclusions of Drs Wei et al. [10]. Previous studies proved estrogen play an important role in lipid metabolism [11]. LDL, TC and TG of postmenopausal women were increased in China [12].There may be unique environmental and socioeconomic factors due to high TG levels in nonelderly with AMI. Changes in diet and lifestyle may be were main factors, which reflect in residents of the area prefer to eat greasy food that increases the risk of dyslipidemia. At the same time, several studies also have shown that elevated TG levels are strongly related to lifestyle factors, which including increased body mass index, lower physical activity levels, increased intake of high calorie foods, and the specific mechanism was unclear [13–15].

According to the diagnostic criteria of dyslipidemia from guidelines for the prevention of dyslipidemia in Chinese adults 2007, Our data indicated that 31.1–40.6% of the nonelderly and 43.1–48% of the elderly AMI patients had normal lipids, and that AMI patients who had LDL levels <80 mg/dL accounted for 23%. Therefore, most of AMI patients require lipid-lowering therapy in the south of China. Studies had shown that early statin treatment reduced mortality, at the same time, it also can significantly improve the prognosis of patients with myocardial infarction who regardless of the level of blood lipids [16, 17]. The main mechanism is that statins are used to delay the progression of coronary atherosclerosis, minimize and stabilize plaques, eventually reducing cardiovascular events. Thus, it is necessary promoted intensive lipid-lowering therapy for AMI patients who had normal lipid files [18].

Our study found that compared with females, males had higher LDL and TG for elderly patients, while contrary to low HDL. LDL-C and HDL-C were considered to produce adverse effects on the risk of myocardial infarction. The level of LDL can be reduced to the low limit approaching normal state (<70 mg/dL) by statin therapies [19]. However, statins do not completely prevent cardiovascular events, which data shown that cardiovascular events were reduced only 25–40% [20, 21]. In addition elevated LDL-C levels, lower level of HDL is strongly associated with cardiovascular events. Although there has been little acceptable treatment to improve HDL levels, these findings have important implications for lipid-lowering therapy in patients with acute myocardial infarction, in which lower HDL levels was associated with increased risk for myocardial infarction even if statin-treated patients who achieve LDL-C < 70 mg/dL.

## Conclusion

In conclusion, our results confirmed that serum lipid levels varied in age and sex in Hakka patients with acute myocardial infarction in southern China. Dyslipidemia is more prevalent in the non-elderly than elderly for males. Levels of TC, LDL, HDL, and TG were higher in females than males for the elderly Hakka population in southern China. Therefore, they may require lipid-lowering therapy to avoid the increased risk of cardiovascular disease. The most common combined dyslipidemia was the isolated high TG for AMI patients, and reduce TG may be critical to such patients.

## Abbreviations

AMI: Acute myocardial infarction
CAD: Coronary artery disease
CHD: Coronary heart disease
CVD: Cardiovascular disease
CVD: Cerebrovascular disease
HDL: High-density lipoprotein cholesterol
LDL: Low-density lipoprotein cholesterol
TC: Total cholesterol
TG: Triglyceride
